# Psychological Endurance, Anxiety, and Coping Style among Journalists Engaged in Emergency Events: Evidence from China

**Published:** 2019-01

**Authors:** Long WANG, Canhua KANG, Zongyi YIN, Fang SU

**Affiliations:** 1. School of Management, China Research Center for Emergency Management, Wuhan University of Technology, Wuhan, China; 2. School of Economics, Wuhan University of Technology, Wuhan, China; 3. School of Arts and Law, Wuhan University of Technology, Wuhan, China

**Keywords:** Emergency events, Journalists, Psychological endurance, Anxiety, Coping style

## Abstract

**Background:**

During reports of emergency events, journalists experience psychological crisis and negative emotions aroused by the events. The psychological endurance of journalists, which is induced by high risks of the career, has attracted widespread attention. Emotional state and coping style are important factors that influence the psychological endurance of individuals. This study aimed to analyze the relationship of psychological endurance with anxiety and coping style among journalists engaged in emergency events.

**Methods:**

A total of 296 journalists in Hubei Province of China who participated in reports on emergency events from August to December 2017 were selected. Journalists were assessed using the Psychological Endurance Scale, Self-rating Anxiety Scale, and Simple Coping Style Questionnaire.

**Results:**

The total score of the psychological endurance of journalists engaged in emergency events is slightly lower than that of the Chinese norm. Score of anxiety of highly educated journalists is significantly higher than that of lowly educated journalists. Psychological endurance of journalists is negatively correlated with negative coping style and anxiety, but it is positively correlated with positive coping style. Positive coping style of journalists engaged in emergency events partially mediates the effects of psychological endurance on anxiety.

**Conclusion:**

Among journalists engaged in emergency events, the highly educated group presents obvious anxiety and negative coping style. Psychological endurance indirectly affects through positive coping style and directly affects anxiety of journalists.

## Introduction

Journalists should be present at the scene of accidents to collect news information in real time. The uncertainty of the working environment in the scene determines the high occupational risk of journalists (1, 2). In particular, journalists face high occupational risks in reporting emergency events. Emergency events differ from general news events due to the former’s uncertain and abrupt nature. Emergency events mainly refer to natural disasters, accidents, and public health and social safety events that occur suddenly, cause serious social harm, and require emergency measures. This scope implies that journalists engaged in emergency events must work in chaotic, noisy, and even dangerous working environment, and they witness and experience traumatic scenes after these emergency events (3). The poor working environment may cause negative emotional experience, and poor psychological endurance further makes journalists develop anxiety, impatience, and other negative psychological features. In addition, the particularity of the job requires journalists who are engaged in emergency events to participate in post treatment and coordination of emergency events for timely and objective reports (4). Increased workload also induces indifference and negative coping styles. Such changes may also indirectly transmit and influence the psychological endurance of journalists. In particular, social responsibility of journalist increases gradually as propagation of social information accelerates (5). Journalists guide the opinion of the public, so they are crucial to promoting the sound development of the media industry and facilitating social progress and harmony. Therefore, the psychological endurance of journalists, especially of those engaged in emergency events, deserves much attention. Existing studies showed that the mood of journalists engaged in emergency events is in a state of anxiety to a certain extent due to the particularity of the working environment of emergency events (6). Anxiety easily causes psychological trauma, which affects the psychological endurance of journalists (7). Moreover, the greater work pressure of emergency news reports compared with that of general news reports increases the challenges and psychological burden of journalists, which makes them change their coping styles (8, 9).

Although existing studies achieved substantial results on psychological endurance, a systematic study on the psychological endurance of journalists engaged in emergency events from the perspective of anxiety and coping style has yet to be conducted. Psychological endurance is an important index that evaluates the working ability of journalists. Evaluation of psychological endurance bears important practical guidance to protect the psychological health of journalists and improve the quality of news reports.

In this study, the relationships of psychological endurance with anxiety and coping style of journalists engaged in emergency events were studied on the basis of the assessment results to reveal the mechanism of the influence. The findings can provide decision-making references in formulating psychological intervention strategies for journalists engaged in emergency events.

## Materials and Methods

### Research Object

A questionnaire survey on the psychological endurance, anxiety, and coping style of journalists from *Hubei Daily*, *Chutian City Newspaper*, *Yangzi River Daily*, and *Wuhan Evening News* in Hubei Province, China who have participated in reports on emergency events was carried out to analyze the relationships of psychological endurance with anxiety and coping style. A total of 356 questionnaires were sent from August to December 2017, and 312 questionnaires were collected. Invalid questionnaires were excluded because of excessive misses and regular distribution of answers. Finally, 296 questionnaires were considered valid, which showed an effective recovery rate of 83.2%.

The investigation was approved by the Ethics Committee of the Yangzi River Daily Press Group, and prior to the investigation, it was necessary for each participant to have informed consent. All methods were performed in accordance with the relevant guidelines and regulations.

Data were analyzed according to the demographic characteristics of the respondents. In terms of gender, 180 male (60.8%) and 116 female (39.2%) respondents comprised the sample. The age range varied from 20–30 (n=104), 31–40 (n=112), 41– 50 (n=44), and over 50 (n=36). The respondents aged less than 40 accounted for 73.0%, indicating that most respondents were young. In terms of education, 20 respondents received junior college education or lower, whereas the remaining 276 respondents have bachelor degrees or higher (93.2%), indicating the high educational level of journalists engaged in emergency events. According to marital status, 107 and 189 respondents were single and married, respectively.

### Research Tools

#### 1) Psychological Endurance Scale

The Psychological Endurance Scale (10), was used as the tool to measure the respondents’ psychological endurance. This scale features a comprehensive consideration to the special psychological features and diversity of Chinese professionals. Therefore, it is superior to other psychological endurance scales in terms of scientificity and applicability to the measurement of psychological endurance in the Chinese population. This scale covers five subscales, namely, Willpower, Family Supports, Optimism & Confidence, Problem Solving, and Interpersonal Communication, which comprises a total of 27 items. A Likert four-point scoring method was adopted that ranged from “completely disagree =1, disagree =2, basically agree =3, and completely agree =4”. All items were individually scored. Respondents with high total scores had strong psychological endurance. Kong LM et al. (10) pointed out that other studies achieved Cronbach’s α coefficient for the *Psychological Endurance Scale* at 0.751 (*p*<0.01), which indicates good internal consistency and high reliability of the scale.

#### 2) Self-rating Anxiety Scale

The Self-rating Anxiety Scale (SAS) was used to measure the anxiety of journalists engaged in emergency events. The SAS was compiled by Zung in 1971 and covered 20 items. It especially emphasizes the subjective experiences of respondents and uses a four-level scoring system that ranged from 1 = no (or rare), 2 = sometimes, 3 = common, and 4 = most of the time (or all the time). Respondents scored themselves according to their feelings. At the end of assessment, the total score of 20 items was multiplied by 1.25 then rounded. The results were used as the standard total score. The respondent is in a normal emotional state when the standard total score is lower than 50, slight anxiety for scores that range from 50 to 59, moderate anxiety in the range of 60–69, and severe anxiety scores over 70. The SAS is a common scale in clinical psychiatry research, which shows relatively positive measurement accuracy (11).

#### 3) Simple Coping Style Questionnaire

Considering various pressure scenarios that journalists may encounter during reporting of emergency events, the Simple Coping Style Questionnaire (SCSQ) was used to assess the attitudes and behaviors of individuals that are faced with stressful events. The SCSQ was compiled according to the characteristics of the Chinese people based on existing scales and has been widely used in relevant studies (12). This questionnaire is composed of two subscales, namely, positive and negative coping styles, which cover 20 items. Specifically, the positive coping style subscale comprises the first to 12th items, and the negative coping style subscale covers the 13th to 20th items. A four-level scoring system was adopted, where 0=no use, 1=occasional use, 2=sometimes use, and 3=frequent use. Respondents scored themselves, in which a high total score reflects a strong positive attitude in coping with pressure.

### Statistical Methods

Data analysis was accomplished by SPSS 21.0 Software (Chicago, IL, USA). Analysis results conforming to normal distribution were expressed by (mean ± standard deviation). *t*-test was used for intergroup comparison. Pearson’s correlation and regression analyses were conducted on the relationship of psychological endurance to coping style and anxiety. *p*<0.05 indicates statistical significance of differences.

## Results

### Analysis of the Assessment Results of Scales

Table 1 provides the assessment results on the psychological endurance of respondents. They were compared with those of the Chinese norm. Scores for Willpower, Family Supports, Optimism & Confidence, Problem Solving, and Interpersonal Communication were used as test variables in the independent sample *t*-test. According to the results, the total score of psychological endurance of journalists engaged in emergency events (79.82±14.39) was significantly lower than that of Chinese adults (*P*<0.05). Specifically, scores of Willpower (16.25±3.36), Family Supports (17.97±3.72), and Optimism & Confidence (17.67±3.17) were significantly lower than those of the Chinese norm (*P*<0.05), whereas Interpersonal Communication (12.32±1.52) was significantly higher (*P*<0.01). However, the score for Problem Solving (15.61±2.62) was higher than that of the Chinese norm, but a statistically non-significant difference was observed. Experience in reports of emergency events can indeed decrease endurance and adaptation of journalists to psychological pressure and negative emotions.

**Table 1:** Assessment results of psychological endurance

***Psychological endurance***	***Journalists engaged in emergency events (n=296)***	***Chinese norm***	***t-test***
Willpower	16.25 ± 3.36	17.42 ± 3.78	1.97 ^*^
Family Supports	17.97 ± 3.72	18.95 ± 3.56	2.11 ^*^
Optimism & Confidence	17.67 ± 3.17	18.11 ± 3.12	2.57 ^*^
Problem Solving	15.61 ± 2.62	15.23 ± 2.52	2.19
Interpersonal Communication	12.32 ± 1.52	12.19 ± 1.59	2.54 ^**^
Total score	79.82 ± 14.39	81.9 ± 14.57	2.39 ^*^

Note:

* *P* <0.05 and

** *P* <0.01

Moreover, individuals’ cognitive sensitivity is influenced by knowledge structure and working experiences. Hence, respondents were divided into two groups by educational background for the *t*-test to reveal differences in anxiety among journalists engaged in emergency events with varying educational backgrounds. Table 2 shows that journalists engaged in emergency events suffered from anxiety in general. Only 52 respondents (17.6%) did not develop anxiety.

**Table 2:** Comparison on anxiety of respondents with different educational backgrounds

***Variable***	***Junior college or lower (n=68)***		***Bachelor degree or higher (n=201)***		***t-value***
	***Proportion***	***Scores***	***Proportion***	***Scores***	
Anxiety-free	32.4% (n=22)	38.27±1.37	14.9% (n=30)	44.19±3.08	2.92 ^*^
Slight anxiety	38.3% (n=26)	53.65±4.33	31.8% (n=64)	58.88±1.28	3.71 ^**^
Moderate anxiety	23.4% (n=16)	62.65±3.33	42.4% (n=85)	65.11±2.28	2.55 ^*^
Severe anxiety	5.9% (n=4)	73.61±1.09	10.9% (n=22)	74.25±4.28	1.98 ^*^

Note:

* *P* < 0.05 and

** *P* < 0.01

In addition, journalists with varying educational backgrounds who are engaged in emergency events showed significant differences in anxiety. Among the anxiety-free and slight, moderate, and severe anxiety respondents, 32.4%, 38.3%, 23.4%, and 5.9%, respectively, achieved junior college or lower education and 14.9%, 31.8%, 42.4%, and 10.9%, respectively, achieved a bachelor degree or higher. By comparing the scores for different items between the two groups, we find that highly educated journalists achieved higher scores than those of lowly educated journalists in term of slight, moderate, and severe anxiety. These results demonstrated that highly educated journalists who have participated in emergency events easily become anxious. Table 3 further reveals the coping style of respondents with different educational backgrounds to setbacks or dilemmas.

**Table 3:** Coping styles of respondents with different education backgrounds

	***Junior college or lower (n=68)***		***Bachelor degree or higher (n=201)***		***t-value***
	* Proportion *	* Scores *	* Proportion *	* Scores *	
Negative coping style	25% (n=17)	2.35±0.39	67.7% (n=136)	2.47±0.42	2.18 ^**^
Positive coping style	75% (n=51)	2.57±0.49	32.3% (n=65)	2.23±0.38	3.01 ^**^

Note:

* *P* < 0.05 and

** *P* < 0.01

According to the results, 25% of respondents with junior college or lower education adopted the negative coping style, whereas 75% adopted the positive one. Besides, 67.7% of respondents with a bachelor degree or higher adopted the negative coping style, whereas only 32.3% adopted the positive one. Moreover, the score of negative coping style of highly educated journalists (2.47±0.42) was higher than that of lowly educated journalists (2.23±0.38). This result demonstrated that highly educated journalists were more negative in the face of difficulties or setbacks in reporting emergency events.

### Correlation Analysis

In Table 4, the correlation analysis results showed that the coping style and anxiety of journalists engaged in emergency events were significantly correlated with psychological endurance. According to the total score, positive coping style was positively correlated with psychological endurance (0.072*), whereas negative coping style was negatively correlated with psychological endurance (−0.123*). Moreover, anxiety was negatively correlated with psychological endurance (−0.299*).

**Table 4:** Correlation of psychological endurance of journalists with coping style and anxiety

***Variable***	***1***	***2***	***3***	***4***	***5***	***6***	***7***	***8***	***9***
1 Willpower	1								
2 Family Supports	0.175 ^**^	1							
3 Optimism & Confidence	0.163 ^*^	0.317 ^*^	1						
4 Problem Solving	0.296 ^**^	0.189 ^**^	0.138	1					
5 Interpersonal Communication	0.357 ^*^	0.428 ^*^	0.267 ^*^	0.221 ^**^	1				
6 Psychological endurance	0.632 ^**^	0.794 ^*^	0.581 ^**^	0.724 ^**^	0.605 ^*^	1			
7 Positive coping	0.078 ^*^	0.046 ^*^	0.055 ^**^	0.079 ^*^	0.008 ^*^	0.072 ^*^	1		
8 Negative coping	−0.217 ^*^	−0.287 ^*^	−0.245 ^*^	−0.233 ^*^	−0.199 ^*^	−0.123 ^*^	−0.093 ^**^	1	
9 Anxiety	−0.231 ^**^	−0.236 ^*^	−0.428 ^**^	−0.361 ^*^	−0.231 ^*^	−0.299 ^*^	−0.174 ^**^	0.343 ^**^	1

Note:

* *P* < 0.05 and

** *P* < 0.01

### Mediating Effect of Positive Coping Style

This study further hypothesized that positive coping style played a mediating role between the psychological endurance and anxiety of journalists engaged in emergency events (Table 5).

**Table 5:** Regression analysis of the mediating effect of positive coping style

***Dependent variable***	***Anxiety***	***Positive coping style***	***Anxiety***

	Step 1	Step 2	Step 3
Independent variable			
Psychological endurance	−0.448 ^**^	0.263 ^**^	−0.391 ^**^
Positive coping style			−0.212 ^**^
ΔR ^ 2 ^	0.203	0.072	0.244
F	359.864 ^**^	107.235 ^**^	230.446 ^**^

Note:

* *P* <0.05 and

** *P* <0.01

Table 5 shows that the psychological endurance of journalists engaged in emergency events had a significantly negative effect on anxiety (−0.448**) and significantly positive effect on positive coping style (0.263**). In step 3, psychological endurance and positive coping style retain their significant effects on anxiety (*P*<0.01). The influence coefficient of psychological endurance was reduced from −0.448 to −0.391.

## Discussion

Based on the analysis, the relationships of psychological endurance of journalists engaged in emergency events with anxiety and coping style were discussed.

First, Table 1 shows that journalists engaged in emergency events achieved a lower total score in psychological endurance than that of the Chinese norm. In addition, scores in Willpower, Family Supports, and Optimism & Confidence were significantly lower than those of the Chinese norm. These results might be related with the nature of the job of journalists engaged in emergency events. Excessive workload, heavy social pressure, and inadequate family investments can essentially influence their psychological health, thus making them anxious and pessimistic. All these factors weaken the psychological endurance of journalists engaged in emergency events. Working long-term in an unpleasant environment will weaken willpower, and thereby reduce the score on Optimism & Confidence. Conversely, these journalists may coordinate and cooperate with different departments during reports or problem solving, which determine high levels of internal communication and problem solving compared with normal levels. On this basis, this study posits that further attention should be paid to the psychological endurance of journalists engaged in emergency events. Considering their weak psychological endurance, journalists must attend essential psychological counseling courses and adjust their psychological states. At the same time, family members should increase concern and support to journalists and provide strong living support.

Second, Table 2 shows that journalists engaged in emergency events were divided into groups according to educational background. Journalists with different educational backgrounds also significantly differed in levels of anxiety. Highly educated journalists achieved significantly higher scores in the levels of anxiety, namely, slight, moderate and severe, than those of lowly educated journalists. This difference can be explained by two reasons: first, highly educated journalists acquired poor practical experiences in handling reports during emergency events and easily lose focus during difficult scenarios, thus increasing their anxiety. Second, Yu SQ (13) demonstrated that a high level of knowledge was conducive to deep perception of events. This notion implied that highly educated journalists are prone to emotional resonance in desolate scenarios, thus significantly increasing anxiety.

Third, Table 3 shows that highly educated journalists achieved lower scores in positive coping style than those with low levels of educational background. On the contrary, highly educated journalists achieved higher scores in negative coping style than those with low educational background. These scores reflect that highly educated journalists were apt to compromise rather than face scenarios directly. This tendency might be brought by various problems that may occur during reporting of emergency events compared with general events, which require journalists to be equipped with rich practical experience and high level of ability in solving actual problems. However, highly educated journalists have typically acquired poor practical experience. Hence, they show preference for negative coping style. According to the abovementioned analysis, the study believes that a news unit should enhance the practical experience of practitioners with high educational background, rational personnel scheduling in reporting of emergency events, strengthen their ability in problem solving, and fundamentally eliminate psychological problems in relation to anxiety caused by work confusion.

Finally, the results in Tables 4 and 5 show that positive coping style was positively correlated with psychological endurance, whereas negative coping style and anxiety were negatively correlated with psychological endurance. In particular, improvement of psychological endurance can reduce the anxiety of journalists engaged in emergency events, and positive coping style plays a partial mediating role between psychological endurance and anxiety. A significant increase in the ability of journalists’ willpower and interpretation, as well as support from family can increase their ability to cope with difficulties and setbacks in the face of emergency events. Therefore, using the positive coping style effectively relieves the anxiety of journalists. An individual’s lack of self-confidence and will to fight is disadvantageous to their working ability and easily causes family conflicts. Journalists engaged in emergency events are likely unable to handle good family relationships and reduce their social skills and problem-solving abilities. Therefore, the lack of psychological endurance is an important reason that causes the anxiety of journalists engaged in emergency events. To enhance psychological endurance of journalists engaged in emergency events, relevant departments should regularly offer special psychological counseling training that aims to improve immunity to unpleasant working environment. Journalists engaged in emergency events not only have to enhance business skills but should also strengthen daily exercises, positively participate in family parties, learn self-adjustment, and ensure adequate sleep. By so doing, they can eliminate pessimistic tendencies, such as depression and anxiety.

## Conclusion

Journalists engaged in emergency events are faced with unprecedented challenges and working pressure due to their special working environment and requirements. Therefore, their psychological endurance has attracted widespread attention. On this basis, 296 journalists from Hubei Province, China engaged in emergency events from August to December 2017 were selected for the questionnaire survey. The relationships of psychological endurance with anxiety and coping style were analyzed. The following conclusions are drawn. The psychological endurance of journalists engaged in emergency events is lower than that of the Chinese norm. The degree of anxiety of highly educated journalists is higher than those with low educational background. In the overall samples, lowly educated journalists prefer the positive coping style during pressure and setbacks, whereas highly educated journalists prefer the negative coping style. Positive coping style of journalists engaged in emergency events plays a partially mediating role in the relationship between psychological endurance and anxiety.

## Ethical considerations

Ethical issues (Including plagiarism, Informed Consent, misconduct, data fabrication and/or falsification, double publication and/or submission, redundancy, etc.) have been completely observed by the authors.
